# Temporal variation of renal function in people with type 2 diabetes mellitus: A retrospective UK clinical practice research datalink cohort study

**DOI:** 10.1111/dom.13734

**Published:** 2019-05-06

**Authors:** Dionysis Spanopoulos, Hajra Okhai, Francesco Zaccardi, Abigail Tebboth, Brendan Barrett, Michael Busse, Joanne Webb, Kamlesh Khunti

**Affiliations:** ^1^ Market Access Boehringer Ingelheim Ltd Bracknell UK; ^2^ Diabetes Research Centre University of Leicester Leicester UK; ^3^ Medical Affairs Boehringer Ingelheim Ltd Bracknell UK; ^4^ Medical Affairs Eli Lilly and Company Basingstoke UK

**Keywords:** primary care, renal impairment, type 2 diabetes, UK

## Abstract

**Aim:**

To characterize the longitudinal variability of estimated glomerular filtration rate (eGFR) in people with type 2 diabetes mellitus (T2DM), including variation between categories and individuals.

**Methods:**

People with T2DM and sufficient recorded serum creatinine measurements were identified from the Clinical Practice Research Datalink (T2DM diagnosis from 1 January 2009 to 1 January 2011 with 5 years follow‐up); eGFR was calculated using the CKD‐EPI equation.

**Results:**

In total, 7766 individuals were included; 32.8%, 50.2%, 12.4%, 4.0% and 0.6% were in glomerular filtration rate (GFR) categories G1, G2, G3a, G3b and G4, respectively. Overall, eGFR decreased by 0.44 mL/min/1.73 m^2^ per year; eGFR increased by 0.80 mL/min/1.73 m^2^ between index and year 1, then decreased by 0.75 mL/min/1.73 m^2^ annually up to year 5. Category G1 showed a steady decline in eGFR over time; G2, G3a and G3b showed an increase between index and year 1, followed by a decline. Category G4 showed a mean eGFR increase of 1.85 mL/min/1.73 m^2^ annually. People in categories G3‐G4 moved across a greater number of GFR categories than those in G1 and G2. Individual patients' eGFR showed a wide range of values (change from baseline at year 5 varied from −80 to +59 mL/min/1.73 m^2^).

**Conclusion:**

Overall, eGFR declined over time, although there was considerable variation between GFR categories and individuals. This highlights the difficulty in prescribing many glucose‐lowering therapies, which require dose adjustment for renal function. The study also emphasizes the importance of regular monitoring of renal impairment in people with T2DM.

## 1 INTRODUCTION

Diabetes is a leading cause of chronic kidney disease (CKD)^1^ and it is expected that between 40% and 50% of people with type 2 diabetes mellitus (T2DM) will be affected by CKD in their lifetimes.^2^, ^3^, ^4^ However, only a small number of glucose‐lowering therapies can be used safely in people with renal impairment without requiring a dose adjustment.^5^ Therefore, renal function is an important factor to consider when prescribing glucose‐lowering medications in people with T2DM.

Previous research has showed that renal function, as measured by estimated glomerular filtration rate (eGFR), can vary considerably, especially among people with diabetes.^6^, ^7^, ^8^, ^9^, ^10^, ^11^, ^12^, ^13^, ^14^ These studies have also suggested that eGFR improvement among people with T2DM is possible,^11^ leading to increased complexity when considering optimal treatment. Published studies have tended to investigate renal variation at the population or category level, with one such study reporting eGFR trends in the UK.^11^ There are no recent studies reporting patient‐level variation in renal function in a T2DM population.

Using primary care clinical records, this study aims to further characterize the longitudinal variability of eGFR in a cohort of people with T2DM with availability of consistent eGFR measurements over a period of 5 years to further explore eGFR trends and patterns over a longer period, including analysis at the individual patient level.

## 2 MATERIALS AND METHODS

### 2.1 Data source

Patient records were obtained from the UK Clinical Practice Research Datalink (CPRD), a primary care database that includes data from general practices throughout the UK. As of November 2018, the database contained anonymized data for approximately 10 million people, with over 1 in 10 practices in the UK contributing data.^15^ CPRD data have been used in over 2000 peer‐reviewed publications^15^, and have been found to be broadly representative of the UK population in terms of age, sex, ethnicity and body mass index (BMI).^16^ Medical records are updated monthly from participating practices, including complete clinical information, pathology tests, anthropometric data, referral and prescription records. CPRD is linked to Hospital Episode Statistics (HES), a database containing details of all hospital admissions, accident and emergency attendances and outpatient appointments, to improve ethnicity recording for glomerular filtration rate (GFR) estimation.^17^

### 2.2 Study population

Individuals were identified in CPRD based on their first diagnosis code of T2DM (codes are reported in the supporting information). Eligibility criteria included diagnosis of T2DM between 1 January 2009 and 1 January 2011; individuals also had to have a measure of serum creatinine after T2DM diagnosis (index measurement) and at least one measure of serum creatinine recorded in 5 yearly intervals post‐first serum creatinine after diagnosis. In addition, the following inclusion criteria were applied: individuals must have at least 12 months' registration in practice prior to the index date; belong to an “up‐to‐standard” practice at the index date; have a record of ethnicity (identified through HES linkage, or CPRD if unavailable in HES). Individuals with a history of type 1 diabetes mellitus were excluded from the analysis.

### 2.3 Renal function classification

Renal function was measured via eGFR using the Chronic Kidney Disease Epidemiology Collaboration (CKD‐EPI) equation. To estimate GFR, the CKD‐EPI equation requires data for serum creatinine, age, sex and ethnicity (see equation in the supporting information).^18^ The CKD‐EPI equation was selected as it is the recommended formula by the National Institute for Heath and Care Excellence (NICE).^19^ Individuals were grouped into GFR categories, as adopted by NICE guidelines, according to their eGFR at baseline and follow‐up.^19^ These are the same categories used by the Kidney Disease: Improving Global Outcomes (KDIGO) CKD Work Group in their international guidelines for the management of CKD.^20^

### 2.4 Data analysis

This was a retrospective, descriptive study. Individuals were grouped into the five clinical categories: G1 (>90 mL/min/1.73 m^2^), G2 (60‐89 mL/min/1.73 m^2^), G3a (45‐59 mL/min/1.73 m^2^), G3b (30‐44 mL/min/1.73 m^2^) and G4 (15‐29 mL/min/1.73 m^2^) based on their renal function at baseline and according to each subsequent yearly measurement. Category G5 (<15 mL/min/1.73 m^2^) was also considered, but none of the study population had an eGFR that fitted within this group.

Baseline characteristics, including age at T2DM diagnosis, age at the index date, BMI, HbA1c, systolic blood pressure, diastolic blood pressure and eGFR, were compared among individuals included and excluded from the analysis using Student's t‐test. Renal function was described for each yearly interval based on the last recorded value per year and compared with baseline using mean values, counts and percentages to identify the raw change in eGFR as well as individual category changes. The analysis was performed using Stata version 14.

## 3 RESULTS

A total of 46 813 people with newly diagnosed T2DM were identified; of these, 7766 (16.6%) met the study inclusion criteria (Figure S1). Most of the included population was white (91.7%), with South Asian, Black, Chinese and other ethnicities accounting for 4.8%, 1.7% and 1.4% of patients, respectively (Table S1). G2 was the most common GFR category, representing 50.2% (3900/7766) of the study population at the index date; 2550 (32.8%), 962 (12.4%), 307 (4.0%) and 47 (0.6%) were in categories G1, G3a, G3b and G4, respectively (Table S1). No patients were in category G5.

Of the 7766 patients included, only a small subset (1037) had a recorded albumin creatinine ratio (ACR). No patients had severely increased ACR (A3; >30 mg/mmol); approximately 24% (253/1037) had moderately increased ACR (A2; 3‐30 mg/mmol) and 76% (784/1037) had normal ACR (A1; <3 mg/mmol).

On average, there were no relevant differences in the baseline characteristics of those included and excluded from the analysis in terms of age, BMI, HbA1c, systolic and diastolic blood pressure and eGFR (Table S1).

### 3.1 Trend analysis

**Figure 1:**
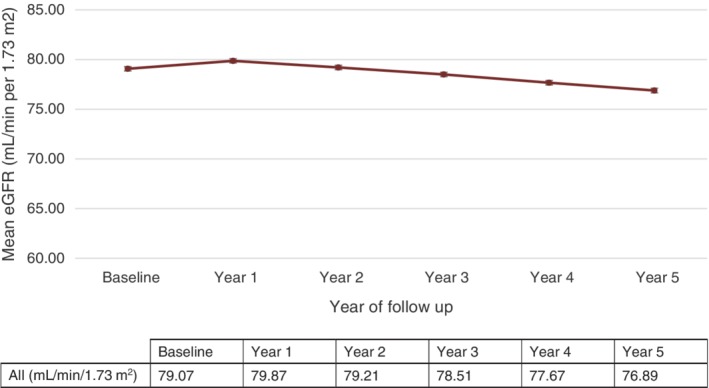
Overall estimated glomerular filtration rate (eGFR) trend

On average, the population's eGFR decreased by 0.44 mL/min/1.73 m^2^ annually. However, an eGFR increase of 0.80 mL/min/1.73 m^2^ was observed between the index measurement and year 1; this was followed by a steady eGFR decline (Figure 1).

**Figure 2:**
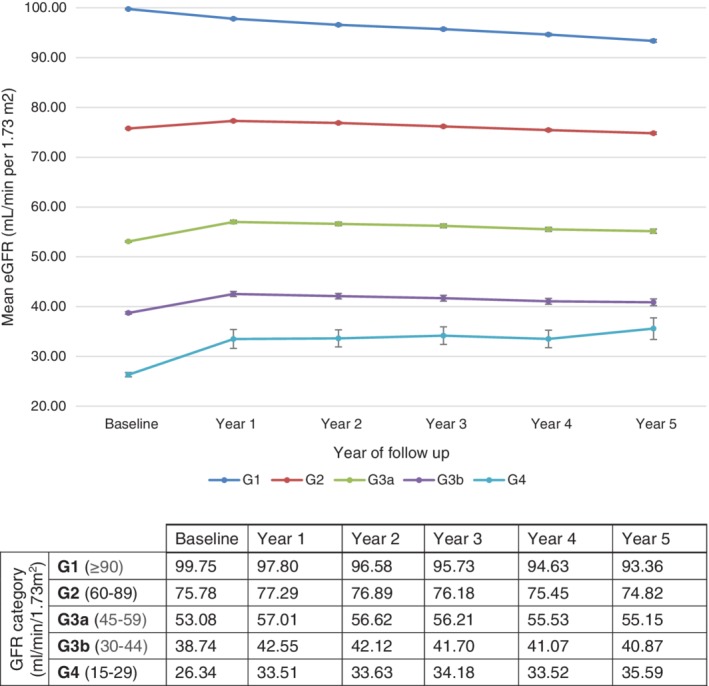
Estimated glomerular filtration rate (eGFR) trend by glomerular filtration rate (GFR) category at baseline. Bars in the graph indicate SD

People in the G1 category at baseline presented with a steady eGFR decline of 1.28 mL/min/1.73 m^2^ annually; those in categories G2, G3a and G3b presented with an eGFR increase between index and year 1, followed by a steady decline; and those in the G4 category showed an overall increase in eGFR of 1.85 mL/min/1.73 m^2^ annually (Figure 2).

### 3.2 GFR category transition analysis

At year 5, 21.3% (1651/7766) of individuals had moved to a lower GFR category and 20% (1042/5216) had moved to a higher GFR category (Table 1). Of the 1316 people in category G3a or below at baseline, 28.3% (373/1316) moved to G2 or above (eGFR ≥60 mL/min/1.73 m^2^) at year 5.

**Table 1:** Glomerular filtration rate (GFR) category transition analysis at the end of follow‐up (year 5)

			GFR category at year 5 (ml/min/1.73 m^2^)								
			G1	G2	G3a	G3b	G4	Increase		Decrease	
			2305 (29.7%)	3891 (50.1%)	975 (12.6%)	496 (6.4%)	99 (1.3%)				
								N	%	N	%
GFR category at baseline (ml/min/1.73 m^2^)	G1 (≥90)	2550 (32.8%)	1726 (67.7%)	763 (29.9%)	41 (1.6%)	17 (0.7%)	3 (0.1%)	N/A	N/A	824	32.3
	G2 (60‐89)	3900 (50.2%)	574 (14.7%)	2760 (70.8%)	454 (11.6%)	104 (2.7%)	8 (0.2%)	574	14.7	566	14.5
	G3a (45‐59)	962 (12.4%)	4 (0.4%)	340 (35.3%)	405 (42.1%)	191 (19.9%)	22 (2.3%)	344	35.8	213	22.1
	G3b (30‐44)	307 (4.0%)	1 (0.3%)	24 (7.8%)	70 (22.8%)	164 (53.4%)	48 (15.6%)	95	30.9	48	15.6
	G4 (15‐29)	47 (0.6%)	0 (0.0%)	4 (8.5%)	5 (10.6%)	20 (42.6%)	18 (38.3%)	29	61.7	0	0.0
								1042	20.0	1651	21.3

Red squares indicate a change to a lower GFR category, whereas green squares indicate a change to a higher GFR category. Yellow squares indicate no change in GFR category.

Abbreviations: eGFR, estimated glomerular filtration rate.

During follow‐up, patients changed GFR categories 1.5 times on average [standard deviation (SD) 1.6]. Those with reduced renal function below 60 mL/min/1.73 m^2^ (G3 and higher categories) changed GFR categories more often compared with people with eGFR ≥60 mL/min/1.73 m^2^ (G1 and G2) (Table 2). In particular, people in categories G1 and G2 changed GFR categories 1.3 times on average (SD 1.6 and 1.5, respectively), and people in categories G3a, G3b and G4 changed GFR categories 2.6 (SD 1.7), 2.1 (SD 1.7) and 2.9 (SD 1.9) times, respectively.

**Table 2:** Glomerular filtration rate (GFR) category transition analysis during follow‐up period

		n	Mean	SD	Min	Max	Median	L‐IQR	U‐IQR
GFR category at baseline (mL/min/1.73m ^2^)	G1 (≥90)	2550	1.3	1.6	0	5	0	0	2
	G2 (60‐89)	3900	1.3	1.5	0	5	1	0	2
	G3a (45‐59)	962	2.6	1.7	0	5	3	1	4
	G3b (30‐44)	307	2.1	1.7	0	5	2	1	3
	G4 (15‐29)	47	2.9	1.9	0	5	3	1	5
	G5 (<15)	N/A	N/A	N/A	N/A	N/A	N/A	N/A	N/A
	All	7766	1.5	1.6	0	5	1	0	3

Abbreviations: eGFR, estimated glomerular filtration rate; L‐IQR, lower interquartile range; max, maximum; min, minimum; SD, standard deviation; U‐IQR, upper interquartile range.

### 3.3 Individual patient analysis

**Figure 3:**
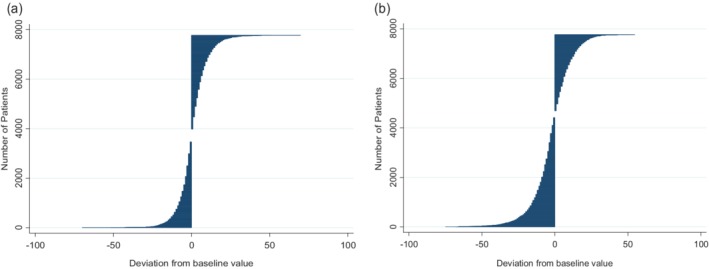
Estimated glomerular filtration rate (eGFR) deviation distribution for (A) year 1 and (B) year 5. Individual level variation represented by line graphs using raw deviation from baseline value for each patient. Each line represents an individual patient's variation from baseline eGFR value, ordered from largest reduction in eGFR to largest increase in eGFR (n = 7766). For example, (B) shows that at year 5, individual patients' difference from baseline eGFR varied from −80 to +59 mL/min/1.73 m^2^

At year 1, 55.5% (4312/7766) of the study population had an increase or no change in their eGFR and 44.5% (3454/7766) had a decrease; 13.6% (1055/7766) of the study population had their eGFR increased by at least 10 mL/min/1.73 m^2^. At year 5, 43.3% (3359/7766) of the study population had an increase or no change in their eGFR and 56.7% (4407/7766) had a decrease; 15.8% (1228/7766) had their eGFR increased by at least 10 mL/min/1.73 m^2^ (Figure 3).

### 3.4 Sensitivity analyses

An analysis of eGFR trends was performed based on the mean of all eGFR values per year, rather than the last recorded eGFR measurement. Overall, this analysis showed that the population's eGFR decreased by 0.45 mL/min/1.73 m^2^ annually compared with 0.44 mL/min/1.73 m^2^ in the main analysis. There was also an increase of 0.65 mL/min/1.73 m^2^ in the first year, followed by a steady decline. Trends in the different GFR categories and at the individual patient level were very similar to those reported in the main analysis (Table S4 and Figure S3, respectively).

A second sensitivity analysis was conducted, using the Modification of Diet in Renal Disease (MDRD) Study equation instead of the CKD‐EPI equation. This analysis also showed a gradual decline in eGFR in the overall population, although it was slower than that shown in the main analysis (0.064 mL/min/1.73 m^2^ annually; Table S4). Change in renal function in the GFR categories and at the individual patient level showed the same trends as reported in the main analysis (Table S4 and Figure S4, respectively).

Finally, we also looked at eGFR trends according to ACR. The results in both categories (A1 and A2) followed a similar trend to that observed in the main analysis (Figure S5).

## 4 DISCUSSION

Although around 50% of people with diabetes develop diabetic nephropathy during their lifetimes,^2^, ^3^, ^4^ disease progression can be improved through early risk factor interventions including glycaemic control^5^ and blood pressure management.^21^ Optimal treatment for people with T2DM is related to renal clearance as only a few glucose‐lowering therapies can be prescribed without consideration of renal function. This study showed that, overall, people with T2DM have a decline in renal function over time; however, there was variation within clinical categories of renal function and at the individual level. In particular, people with worse renal function (eGFR < 60 mL/min/1.73 m^2^) appeared to show the greatest variation, both from the overall study population and from their baseline eGFR. Individuals in the highest GFR category (G1) experienced a consistent and steady decline in renal function over the study period, whereas those in the G2, G3a and G3b categories showed an increase at year 1 followed by decline; lastly, those in the G4 category showed an overall improvement over time. Individuals with an eGFR <60 mL/min/1.73 m^2^ at baseline (G3a, G3b and G4) changed GFR categories more often than those in the higher categories. In addition, around 28% of people with eGFR <60 mL/min/1.73 m^2^ at baseline had an increase in their eGFR to ≥60 mL/min/1.73 m^2^ by year 5. While the change at the overall population level and in some GFR categories was small, there was a greater and more clinically relevant variation at the individual level, with around 15% of patients experiencing an increase of at least 10 mL/min/1.73 m^2^. At year 5, the difference from baseline eGFR varied from −80 to +59 mL/min/1.73 m^2^. This variation highlights the difficulty in making treatment decisions based on a single eGFR estimate, as well as reinforcing the need for regular renal function screening in people with T2DM. This is in line with current UK guidelines for renal monitoring.^19^

This was a retrospective, observational study to describe how renal function varies over time in a contemporary real‐world cohort of people with T2DM; the aim was not to investigate the cause(s) of variation. A number of variables were not assessed in this study, including use of background medication, treatment received for renal impairment, comorbidities and other lifestyle factors that could affect individuals' renal function. Differences in background medications, in particular, could explain some of the variation at category and individual levels, as this could be expected to be different between individuals and GFR categories. For example, all GFR categories except G1 showed an increase in eGFR between index and year 1. This could be because of individuals receiving treatments that affect kidney function [i.e. angiotensin‐converting enzyme (ACE) inhibitors] or a result of modifications in lifestyle factors such as diet, with the effects of these interventions reducing over time, leading to decline in renal function between year 1 and year 5.

Although the study was not designed to explain the causes of variation in renal function, it does highlight the importance of monitoring individuals' renal function when considering their T2DM treatment. It also reflects the situation in the real world where individuals' eGFR can be affected by a number of factors that are not always clear or known. However, there are some limitations to this analysis. The study design may also have contributed to the increasing trend in eGFR for patients in the lower category (G4), because five serum creatinine measurements were required at yearly intervals following the index measurement. This criterion was needed to ensure that patients had enough data to allow sufficient follow‐up, although it is possible that this could have led to the exclusion of individuals with low renal function who died over the study period thereby leaving a sample that overrepresented the G4 patients whose renal function increased over time. The majority of people were excluded because of a lack of serum creatinine measurements during the follow‐up period (82.9%; 32 358 out of 39 047 excluded patients), although analysis showed that there were no relevant differences in the baseline characteristics between included versus excluded individuals.

The CKD‐EPI equation was used in the main analysis as it is the method recommended by NICE for GFR estimation.^19^ NICE recommend the CKD‐EPI equation, as it is considered to be more accurate than the MDRD Study equation at the population level, less biased at a GFR of >60 mL/min/1.73 m^2^, and performs better in people aged 75 years and over.^19^ However, it has been found to lack precision at the individual level.^22^, ^23^ Therefore, we also performed a sensitivity analysis using the MDRD equation to assess the robustness of the results. This analysis showed similar overall trends to the main analysis: a gradual decline in eGFR in the overall population with variation at GFR category and individual patient level. It is also worth considering that most clinicians use estimated, not measured, GFR to manage their patients, despite the limitations in providing patient‐level precision. The methodology of this study is therefore reflective of that carried out in clinical practice, thus the results should be applicable and relevant to real‐world management of patients, particularly in the UK.

As for all CPRD or database studies, the results are dependent on the quality of the data entry. Ethnicity data, for example, are not well recorded in CPRD. HES data were used to supplement the information provided in CPRD, as ethnicity is required to calculate eGFR according to the CKD‐EPI equation. It is possible that use of HES data could increase the proportion of patients with more severe disease or complications than the general population, as these patients are more likely to have an HES record than patients with milder disease. In theory this could lead to a bias in the overall population. However, it should not have a significant impact on the trends shown in GFR categories, or at the individual level. Another measure that is difficult to assess in CPRD is quality of general practitioner or general practice, and this may have an impact on renal function variation in people with T2DM. This may also produce some bias in the results, as we are unable to identify how the practices included in our study perform against any clinical quality metrics. However, because all the practices included met the “up‐to‐standard” metric, it is probable that each possessed a reasonable level of quality and were suitable for research.

Previous research, including observational studies and a randomized controlled trial, has shown that eGFR tends to decline over time among patients with T2DM; some studies have found that groups of patients may experience varying rates of renal function decline, with some exhibiting rapid decline and others slower decline.^6^, ^7^, ^8^, ^9^, ^10^, ^11^, ^12^, ^13^, ^14^ The observational studies, in particular, have also reported differences in the rate of eGFR change based on certain patient characteristics including age, ethnicity, positive or negative proteinuria at baseline and hypertension.^8‐11^ However, no recent studies have reported individual patient‐level variation in renal function in individuals with T2DM. One previous UK study of eGFR change in people with T2DM has used the CPRD database, although it only included people with diagnoses or test results consistent with renal impairment.^11^ Similar to our study, Cid Ruzafa et al. noted that individuals moved up and down eGFR categories between baseline and end of follow‐up.^11^ The investigators also modelled eGFR change as a continuous variable over 5.8 years, taking into account all observed eGFR values during the study period. This showed an overall decrease in eGFR across the whole population, a result that is comparable with the trend observed in our study population. However, the model did not show any particular differences by GFR category, with all groups apart from G3a showing a slow decline in renal function over time. Our analysis used observational data to describe trends in eGFR over time and found that eGFR change from baseline varies across different GFR categories. Our results also highlighted considerable differences in how renal function changes over time at the individual level, which has not been reported previously. Both studies show an overall decreasing trend in renal function for people with T2DM, however, there are some differences in categories and individual patient level data between the analyses, which further highlights the complexity of managing these patients in clinical practice. We also identified a large proportion of the T2DM patient population who did not have yearly records of serum creatinine measurements throughout the study period (that was the reason for exclusion for 82.9% of excluded patients). This gap between real‐world practice and clinical guidelines does not help to reduce the complexity of managing patients with T2DM. Rather, it highlights the importance of a regular assessment of renal function in people with T2DM, also in view of the level of variation in eGFR seen in our study.

*In conclusion*, the results of this study show that, although the overall population experienced a downward trend in their eGFR over time, there was also considerable variation within clinical categories of renal function and at the individual level, with patients' renal function increasing as well as decreasing over 5 years. This highlights the difficulty when prescribing glucose‐lowering therapies based on a single measurement of renal function, as well as the importance of regular monitoring of renal function in people with T2DM.

## CONFLICT OF INTEREST

A.T. and M.B. are employees of Boehringer Ingelheim, the study sponsor. D.S. and B.B. were also employees of Boehringer Ingelheim at the time the study was conducted. J.W. is an employee of Eli Lilly and Company. H.O. and F.Z. received funding from the study sponsors for the analysis and conduct of the study. K.K. has acted as a consultant and or speaker for Napp, Novartis, Novo Nordisk, Sanofi‐Aventis, Lilly and Merck Sharp & Dohme, and has received grants in support of investigator and investigator‐initiated trials from Novartis, Novo Nordisk, Sanofi‐Aventis, Lilly, Pfizer, Boehringer Ingelheim and Merck Sharp & Dohme.

## AUTHOR CONTRIBUTIONS

D.S., H.O., F.Z., B.B., M.B. and J.W. contributed to the plan and design of the study. H.O. and F.Z. completed the data analysis and all authors provided data interpretation. A.T. and D.S. drafted the manuscript, which was critically revised by all other authors. K.K. is a guarantor of this work.

## Supporting information


**Table S1** Baseline characteristics of included and excluded patients
**Table S2**. Read codes used for type 2 diabetes mellitus
**Table S3**. Distribution of eGFR deviation from baseline, at year 1 and year 5
**Table S4**. eGFR trend by GFR category at baseline (sensitivity analyses vs. main analysis)
**Figure S1**. Patient flow diagram
**Figure S2**. CKD‐EPI equation
**Figure S3**. eGFR deviation distribution (sensitivity analysis 1 vs. main analysis)
**Figure S4**. eGFR deviation distribution (sensitivity analysis 2 vs. main analysis)
**Figure S5**. eGFR trends based on ACR category at baseline
