# Supplementary material for: Temporal variation of renal function in people with type 2 diabetes mellitus: A retrospective UK clinical practice research datalink cohort study
Source: Diabetes Obes Metab. 2019 May 6;21(8):1817–23. doi: 10.1111/dom.13734 (PMC6767485; doi:10.1111/dom.13734)
Supplement: Supplementary file 1 — Table S1 Baseline characteristics of included and excluded patients Table S2. Read codes used for type 2 diabetes mellitus Table S3. Distribution of eGFR deviation from baseline, at year 1 and year 5 Table S4. eGFR trend by GFR category at baseline (sensitivity analyses vs. main analysis) Figure S1. Patient flow diagram Figure S2. CKD‐EPI equation Figure S3. eGFR deviation distribution (sensitivity analysis 1 vs. main analysis) Figure S4. eGFR deviation distribution (sensitivity analysis 2 vs. main analysis) Figure S5. eGFR trends based on ACR category at baseline [file DOM-21-1817-s001.docx]

**Supplementary Information**

**Contents**

[Table S1: Baseline characteristics of included and excluded patients 2](#_Toc2671536)

[Table S2: Read codes used for type 2 diabetes mellitus 3](#_Toc2671537)

[Table S3: Distribution of eGFR deviation from baseline, at year 1 and year 5 5](#_Toc2671538)

[Table S4: eGFR trend by GFR category at baseline (sensitivity analyses vs. main analysis) 6](#_Toc2671539)

[Figure S1: Patient flow diagram 7](#_Toc2671540)

[Figure S2: CKD-EPI equation 8](#_Toc2671541)

[Figure S3: eGFR deviation distribution (sensitivity analysis 1 vs. main analysis) 8](#_Toc2671542)

[Figure S4: eGFR deviation distribution (sensitivity analysis 2 vs. main analysis) 9](#_Toc2671543)

[Figure S5: eGFR trends based on ACR category at baseline 9](#_Toc2671544)

# Table S1: Baseline characteristics of included and excluded patients

| **Characteristics** | **Included patients** | **Excluded patients** | **P value** |
| --- | --- | --- | --- |
| Ethnicity, N (%)  Total  White  Mixed  South Asian  Black  Chinese and other  Unknown* | 7,766 (100.0)  7,120 (91.7)  36 (0.5)  372 (4.8)  130 (1.7)  108 (1.4)  0 (0.0) | 39,047 (100.0)  13,639 (34.9)  80 (0.2)  940 (2.4)  412 (1.1)  258 (0.7)  23,718 (60.7) | Not Applicable* |
| GFR category, N (%)  Total  G1  G2  G3a  G3b  G4  G5  Unknown* | 7,766 (100.0)  2,550 (32.8)  3,900 (50.2)  962 (12.4)  307 (4.0)  47 (0.6)  0 (0.0)  0 (0.0) | 39,047 (100.0)  4,959 (12.7)  7,138 (18.3)  1,720 (4.4)  632 (1.6)  132 (0.3)  0 (0.0)  24,466 (62.7) | Not Applicable* |
| Gender, N (%)  Total  Male  Unknown | 7,766 (100.0)  4,361 (56.2)  0 (0.0) | 39,047 (100.0)  22,198 (56.8)  1 (0.0) | Not Applicable* |
| Age at diagnosis in years, mean (SD)  Total with data, N (%) | 62.4 (12.2)  7,766 (100.0) | 60.4 (14.7)  38,995 (99.9) | <0.0001 |
| Age at first serum creatinine test in years, mean (SD)  Total with data, N (%) | 62.8 (12.2)  7,766 (100.0) | 61.1 (14.3)  31,984 (81.9) | <0.0001 |
| BMI in kg/m^2^, mean (SD)  Total with data, N (%) | 29.8 (5.6)  7,756 (99.9) | 32.0 (6.8)  31,787 (81.4) | <0.0001 |
| HbA1c in mmol/mol, mean (SD)  Total with data, N (%) | 53.3 (14.7)  7,714 (99.3) | 56.8 (17.4)  25,452 (65.2) | <0.0001 |
| Systolic blood pressure in mm/Hg, mean (SD)  Total with data, N (%) | 136.0 (16.6)  7,741 (99.7) | 137.4 (17.9)  33,461 (85.7) | <0.0001 |
| Diastolic blood pressure in mm/Hg, mean (SD)  Total with data, N (%) | 78.8 (10.1)  7,741 (99.7) | 80.6 (10.9)  33,461 (85.7) | <0.0001 |
| eGFR in mL/min/1.72m^2^*, mean (SD)  Total with data, N (%) | 79.1 (19.3)  7,766 (100.0) | 79.7 (20.4)  14,581 (37.3) | 0.0396 |

Abbreviations: BMI, body mass index; eGFR, estimated glomerular filtration rate; HbA1c, glycated haemoglobin; SD, standard deviation; T2DM, type 2 diabetes mellitus

*Only patients with a recorded ethnicity were eligible for this study, so that estimated GFR category could be calculated

# Table S2: Read codes used for type 2 diabetes mellitus

| **medcode** | **Readcode** | **Read term** |
| --- | --- | --- |
| 758 | C10F.00 | Type 2 diabetes mellitus |
| 506 | C100112 | Non-insulin dependent diabetes mellitus |
| 4513 | C109.00 | Non-insulin dependent diabetes mellitus |
| 17859 | C109.12 | Type 2 diabetes mellitus |
| 1407 | C10FJ00 | Insulin treated Type 2 diabetes mellitus |
| 18219 | C109.13 | Type II diabetes mellitus |
| 18390 | C10FM00 | Type 2 diabetes mellitus with persistent microalbuminuria |
| 5884 | C109.11 | NIDDM - Non-insulin dependent diabetes mellitus |
| 18278 | C109J00 | Insulin treated Type 2 diabetes mellitus |
| 26054 | C10FL00 | Type 2 diabetes mellitus with persistent proteinuria |
| 12640 | C10FC00 | Type 2 diabetes mellitus with nephropathy |
| 22884 | C10F.11 | Type II diabetes mellitus |
| 18496 | C10F600 | Type 2 diabetes mellitus with retinopathy |
| 8403 | C109700 | Non-insulin dependant diabetes mellitus - poor control |
| 25627 | C10F700 | Type 2 diabetes mellitus - poor control |
| 32627 | C10FN00 | Type 2 diabetes mellitus with ketoacidosis |
| 34912 | C109400 | Non-insulin dependent diabetes mellitus with ulcer |
| 47954 | C10F900 | Type 2 diabetes mellitus without complication |
| 34450 | C10FK00 | Hyperosmolar non-ketotic state in type 2 diabetes mellitus |
| 36695 | C10D.00 | Diabetes mellitus autosomal dominant type 2 |
| 29979 | C109900 | Non-insulin-dependent diabetes mellitus without complication |
| 34268 | C10F200 | Type 2 diabetes mellitus with neurological complications |
| 53392 | C10F911 | Type II diabetes mellitus without complication |
| 18777 | C10F000 | Type 2 diabetes mellitus with kidney complications |
| 35385 | C10FH00 | Type 2 diabetes mellitus with neuropathic arthropathy |
| 25591 | C10FQ00 | Type 2 diabetes mellitus with exudative maculopathy |
| 17262 | C109600 | Non-insulin-dependent diabetes mellitus with retinopathy |
| 18425 | C10FB00 | Type 2 diabetes mellitus with polyneuropathy |
| 60796 | C10FL11 | Type II diabetes mellitus with persistent proteinuria |
| 49074 | C10F400 | Type 2 diabetes mellitus with ulcer |
| 41389 | C105100 | Diabetes mellitus, adult onset, + ophthalmic manifestation |
| 63762 | C10z100 | Diabetes mellitus, adult onset, + unspecified complication |
| 44982 | C10FE00 | Type 2 diabetes mellitus with diabetic cataract |
| 50609 | L180600 | Pre-existing diabetes mellitus, non-insulin-dependent |
| 59365 | C109C00 | Non-insulin dependent diabetes mellitus with nephropathy |
| 46917 | C10FD00 | Type 2 diabetes mellitus with hypoglycaemic coma |
| 47321 | C10F100 | Type 2 diabetes mellitus with ophthalmic complications |
| 62674 | C10FA00 | Type 2 diabetes mellitus with mononeuropathy |
| 47315 | C10F711 | Type II diabetes mellitus - poor control |
| 18264 | C109J12 | Insulin treated Type II diabetes mellitus |
| 24458 | C109711 | Type II diabetes mellitus - poor control |
| 12736 | C10F500 | Type 2 diabetes mellitus with gangrene |
| 36633 | C109K00 | Hyperosmolar non-ketotic state in type 2 diabetes mellitus |
| 63690 | C10FR00 | Type 2 diabetes mellitus with gastroparesis |
| 64668 | C10FJ11 | Insulin treated Type II diabetes mellitus |
| 45913 | C109712 | Type 2 diabetes mellitus - poor control |
| 51756 | C10FP00 | Type 2 diabetes mellitus with ketoacidotic coma |
| 58604 | C109611 | Type II diabetes mellitus with retinopathy |
| 49655 | C10F611 | Type II diabetes mellitus with retinopathy |
| 37806 | C10FF00 | Type 2 diabetes mellitus with peripheral angiopathy |
| 59253 | C10FG00 | Type 2 diabetes mellitus with arthropathy |
| 37648 | C109J11 | Insulin treated non-insulin dependent diabetes mellitus |
| 55075 | C109411 | Type II diabetes mellitus with ulcer |
| 52303 | C109000 | Non-insulin-dependent diabetes mellitus with kidney comps |
| 42762 | C109612 | Type 2 diabetes mellitus with retinopathy |
| 24836 | C109C12 | Type 2 diabetes mellitus with nephropathy |
| 45467 | C109B00 | Non-insulin dependent diabetes mellitus with polyneuropathy |
| 45919 | C109212 | Type 2 diabetes mellitus with neurological complications |
| 50429 | C109100 | Non-insulin-dependent diabetes mellitus with ophthalm comps |
| 69278 | C109E00 | Non-insulin depend diabetes mellitus with diabetic cataract |
| 40401 | C109500 | Non-insulin dependent diabetes mellitus with gangrene |
| 43785 | C109D00 | Non-insulin dependent diabetes mellitus with hypoglyca coma |
| 44779 | C109E12 | Type 2 diabetes mellitus with diabetic cataract |
| 47816 | C109H11 | Type II diabetes mellitus with neuropathic arthropathy |
| 55842 | C109200 | Non-insulin-dependent diabetes mellitus with neuro comps |
| 18209 | C109012 | Type 2 diabetes mellitus with kidney complications |
| 48192 | C109E11 | Type II diabetes mellitus with diabetic cataract |
| 50225 | C109011 | Type II diabetes mellitus with kidney complications |
| 62107 | C109511 | Type II diabetes mellitus with gangrene |
| 65267 | C10F300 | Type 2 diabetes mellitus with multiple complications |
| 65704 | C109412 | Type 2 diabetes mellitus with ulcer |
| 43227 | C10F311 | Type II diabetes mellitus with multiple complications |
| 46150 | C109512 | Type 2 diabetes mellitus with gangrene |
| 64571 | C109C11 | Type II diabetes mellitus with nephropathy |
| 66965 | C109H12 | Type 2 diabetes mellitus with neuropathic arthropathy |
| 24693 | C109G00 | Non-insulin dependent diabetes mellitus with arthropathy |
| 54899 | C109F11 | Type II diabetes mellitus with peripheral angiopathy |
| 61071 | C109D12 | Type 2 diabetes mellitus with hypoglycaemic coma |
| 67905 | C109211 | Type II diabetes mellitus with neurological complications |
| 47409 | C109B11 | Type II diabetes mellitus with polyneuropathy |
| 50527 | C10FB11 | Type II diabetes mellitus with polyneuropathy |
| 57278 | C10F011 | Type II diabetes mellitus with kidney complications |
| 59725 | C109111 | Type II diabetes mellitus with ophthalmic complications |
| 60699 | C109F12 | Type 2 diabetes mellitus with peripheral angiopathy |
| 62146 | C109300 | Non-insulin-dependent diabetes mellitus with multiple comps |
| 72320 | C109A00 | Non-insulin dependent diabetes mellitus with mononeuropathy |
| 18143 | C109G11 | Type II diabetes mellitus with arthropathy |
| 49869 | C109G12 | Type 2 diabetes mellitus with arthropathy |
| 50813 | C109A11 | Type II diabetes mellitus with mononeuropathy |
| 56268 | C109D11 | Type II diabetes mellitus with hypoglycaemic coma |
| 70316 | C109112 | Type 2 diabetes mellitus with ophthalmic complications |
| 85991 | C10FM11 | Type II diabetes mellitus with persistent microalbuminuria |
| 91646 | C10F411 | Type II diabetes mellitus with ulcer |
| 93727 | C10FE11 | Type II diabetes mellitus with diabetic cataract |

Abbreviations: T2DM, type 2 diabetes mellitus

# Table S3: Distribution of eGFR deviation from baseline, at year 1 and year 5

|  | **Year 1** | **Year 5** |
| --- | --- | --- |
| Difference to baseline  (mL/min/1.73m^2^) | N (%) | N (%) |
| -80 to -71 | #N/A | 1 (<1%) |
| -70 to -61 | 1 (<1%) | 11 (0.1%) |
| -60 to -51 | 1 (<1%) | 13 (0.2%) |
| -50 to -41 | 5 (0.1%) | 40 (0.5%) |
| -40 to -31 | 9 (0.1%) | 128 (1.6%) |
| -30 to -21 | 91 (1.2%) | 348 (4.5%) |
| -20 to -11 | 507 (6.5%) | 1096 (14.1%) |
| -10 to -1 | 2840 (36.6%) | 2770 (35.7%) |
| 0 to 9 | 3257 (41.9%) | 2131 (27.4%) |
| 10 to 19 | 838 (10.8%) | 890 (11.5%) |
| 20 to 29 | 165 (2.1%) | 274 (3.5%) |
| 30 to 39 | 40 (0.5%) | 50 (0.6%) |
| 40 to 49 | 10 (0.1%) | 10 (0.1%) |
| 50 to 59 | 1 (<1%) | 4 (0.1%) |
| 60 to 69 | #N/A | #N/A |
| 70 to 79 | 1 (<1%) | #N/A |
| 80 to 89 | #N/A | #N/A |
| 90+ | #N/A | #N/A |

# Table S4: eGFR trend by GFR category at baseline (sensitivity analyses vs. main analysis)

|  | | **Baseline** | **Year 1** | **Year 2** | **Year 3** | **Year 4** | **Year 5** |
| --- | --- | --- | --- | --- | --- | --- | --- |
|  |  | **Main analysis** | | | | | |
| GFR category (ml/min/1.73m^2^) | **G1** (≥90) | 99.75 | 97.80 | 96.58 | 95.73 | 94.63 | 93.36 |
|  | **G2** (60-89) | 75.78 | 77.29 | 76.89 | 76.18 | 75.45 | 74.82 |
|  | **G3a** (45-59) | 53.08 | 57.01 | 56.62 | 56.21 | 55.53 | 55.15 |
|  | **G3b** (30-44) | 38.74 | 42.55 | 42.12 | 41.70 | 41.07 | 40.87 |
|  | **G4** (15-29) | 26.34 | 33.51 | 33.63 | 34.18 | 33.52 | 35.59 |
|  | **All** | 79.07 | 79.87 | 79.21 | 78.51 | 77.67 | 76.89 |
|  |  | **Sensitivity analysis 1 (average eGFR per year)** | | | | | |
| GFR category (ml/min/1.73m^2^) | **G1** (≥90) | 99.75 | 97.95 | 96.83 | 96.03 | 94.75 | 93.48 |
|  | **G2** (60-89) | 75.78 | 77.10 | 76.90 | 76.14 | 75.35 | 74.72 |
|  | **G3a** (45-59) | 53.08 | 56.43 | 56.53 | 55.87 | 55.38 | 54.78 |
|  | **G3b** (30-44) | 38.74 | 41.76 | 41.78 | 41.46 | 41.16 | 40.78 |
|  | **G4** (15-29) | 26.34 | 32.50 | 33.31 | 34.08 | 33.42 | 34.24 |
|  | **All** | 79.07 | 79.72 | 79.27 | 78.54 | 77.64 | 76.82 |
|  |  | **Sensitivity analysis 2 (MDRD equation)** | | | | | |
| GFR category (ml/min/1.73m^2^) | **G1** (≥90) | 102.74 | 99.12 | 98.53 | 97.63 | 96.57 | 95.52 |
|  | **G2** (60-89) | 74.63 | 76.76 | 76.56 | 76.34 | 76.17 | 75.88 |
|  | **G3a** (45-59) | 53.19 | 57.09 | 57.22 | 57.07 | 56.54 | 56.58 |
|  | **G3b** (30-44) | 38.71 | 43.19 | 42.90 | 42.68 | 42.17 | 42.38 |
|  | **G4** (15-29) | 26.16 | 33.22 | 33.68 | 34.85 | 33.90 | 36.34 |
|  | **All** | 76.52 | 77.65 | 77.40 | 77.04 | 76.58 | 76.20 |

# Figure S1: Patient flow diagram


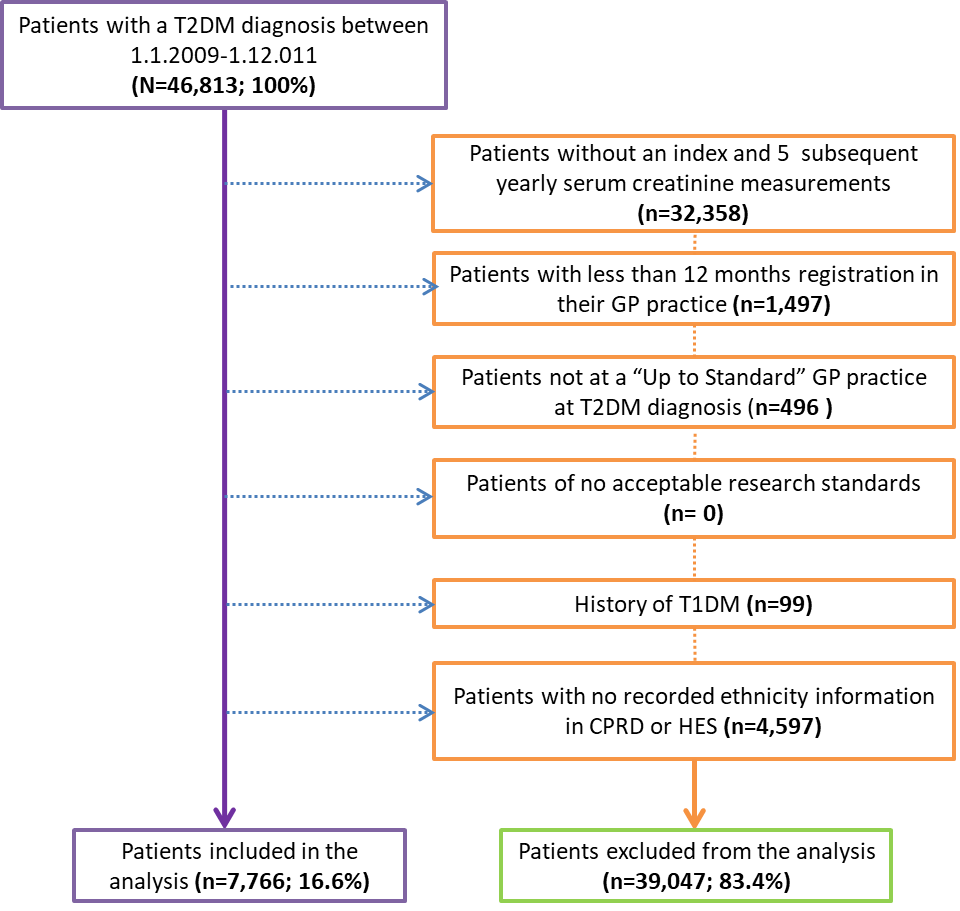


*

*

Abbreviations: CPRD, Clinical Practice Research Datalink; GP, general practitioner; HES, Hospital Episode Statistics; T1DM, type 1 diabetes mellitus; T2DM, type 2 diabetes mellitus

* “Acceptable research standards” and “up to standard” are quality metrics provided by CPRD to give a measure of the suitability of patient records for research. “Acceptable research standards” is a patient metric based on registration status, recording of events in the patient record, and valid age and gender. The “up to standard” date is a practice-based quality metric based on the continuity of recording and the number of recorded deaths, and is calculated for each participating practice according to the latest date at which these minimum quality criteria were met. Whilst these metrics do not guarantee data quality, CPRD recommends they are used as a first step to select research-quality patients for studies.

# Figure S2: CKD-EPI equation

| GFR = 141 x min(Scr/κ, 1)^α^ x max(Scr/κ, 1)^-1.209^ x 0.993^Age^ x 1.018 [if female] x 1.159 [if black],  where Scr is serum creatinine  κ is 0.7 for females and 0.9 for males  α is -0.329 for females and -0.411 for males,  min indicates the minimum of Scr/κ or 1  max indicates the maximum of Scr/κ or 1 |
| --- |

# Figure S3: eGFR deviation distribution (sensitivity analysis 1 vs. main analysis)

a)

b)

(a) year 1; (b) year 5

Abbreviations: eGFR, estimated glomerular filtration rate

Sensitivity analysis 1: average eGFR per year

# Figure S4: eGFR deviation distribution (sensitivity analysis 2 vs. main analysis)

a)

b)

(a) year 1; (b) year 5

Abbreviations: eGFR, estimated glomerular filtration rate

Sensitivity analysis 2: MDRD equation

# Figure S5: eGFR trends based on ACR category at baseline

Abbreviations: ACR, albumin creatinine ratio; eGFR, estimated glomerular filtration rate
